# Effects of Blood Flow Restriction on Leukocyte Profile and Muscle Damage

**DOI:** 10.3389/fphys.2020.572040

**Published:** 2020-10-09

**Authors:** Leandro dos Santos, Michely V. Andreatta, Victor M. Curty, Wena Dantas Marcarini, Lucas G. Ferreira, Valerio G. Barauna

**Affiliations:** ^1^Department of Physiological Sciences, Federal University of Espirito Santo, Vitoria, Brazil; ^2^Academic Unity of Serra Talhada, Rural Federal University of Pernambuco, Serra Talhada, Brazil; ^3^Center of Physical Education and Sports, Federal University of Espirito Santo, Vitoria, Brazil

**Keywords:** Kaatsu training, exercise-induced muscle damage, lymphocytosis, neutrophils, monocytes

## Abstract

Muscle damage affects the blood leukocyte profile. Resistance exercise (RE) with blood flow restriction (BFR) attenuates exercise-induced muscle damage (EIMD).

**Purpose:**

To evaluate muscle damage and the leukocyte profile in response to RE+BFR and to compare with high intensity RE.

**Methods:**

Twenty volunteers performed the RE in the leg press apparatus in the following groups: RE80, 80% of 1RM (3 × until concentric muscle failure); RE40+BFR, 40% of 1RM with BFR (same total work of RE80 group). The BFR applied was 80% of the total occlusion pressure.

**Results:**

There were no differences in the blood leukocyte profile among groups despite the lower exercise-induced muscle damage (EIMD) in the RE40+BFR group (RE80: 10.07 ± 2.67 vs. RE40+BFR: 8.25 ± 0.96; cell × 10^3^/mm^3^). Both groups showed leukocytosis (RE80: 7.59 ± 1.48 vs. 10.07 ± 2.67 and RE40+BFR: 6.57 ± 1.50 vs. 8.25 ± 0.96; cell × 10^3^/mm^3^) and lymphocytosis (RE80: 2.48 ± 0.83 vs. 3.65 ± 1.31 and RE40+BFR: 2.22 ± 0.23 vs. 3.03 ± 0.65; cell × 10^3^/mm^3^) immediately after exercise. Leukocytosis (ES 1.12 vs. ES 1.33) and lymphocytosis (ES 1.11 vs. ES 1.76) was greater in the RE40+BFR group.

**Conclusion:**

RE associated with BFR was accompanied by a greater leukocytosis and lymphocytosis immediately after exercise, with no difference in neutrophils. This leukocyte blood profile may be related to less muscle damage, as well as faster muscle recovery after 24 and 48 h post-exercise.

## Introduction

The understanding of the changes in the leukocyte profile in face of different types of acute and chronic exercises has been frequently investigated in healthy and in clinical populations. While evidence suggests that chronic exercise has an anti-inflammatory effect, the impact of acute exercise needs to be better understood (Schlagheck et al., 2020). Leukocytosis is often associated with infection and/or inflammation. However, it is also present in the exercise as a transitory phenomenon, returning to pre-exercise levels between 6 to 24 h after its end (Simpson et al., 2015).

Muscle damage affects the blood leukocyte profile (Peake et al., 2017). Studies demonstrated the mobilization of neutrophils and monocytes after exercise (Peake et al., 2005; Neubauer et al., 2008). Lymphocytes are also mobilized with exercise, however, during the initial stages of recovery, lymphocytopenia occurs (Simpson et al., 2015). This would be linked to greater vulnerability to disease during the recovery phase of exercise, which is known as “open window” (Simpson et al., 2015). These changes have different magnitudes depending on the type, intensity and duration of the exercise, as well as physical fitness, age and other variables (Hulmi et al., 2010). In contrast, a more recent interpretation of the lymphocytopenia has been raised. It has been showed that lymphocytes are redistributed to potential sites of infection rather than a real decrease and thus would actually be related to a better immune response (Campbell and Turner, 2018). Recent studies have demonstrated that accumulation of these cells in the injured muscle has a fundamental role in its repair and regeneration post-exercise (Peake et al., 2017; Deyhle and Hyldahl, 2018).

Exercise-induced muscle damage (EIMD) is molecularly characterized by transient ultrastructural myofibrillar disruption and efflux of myocellular enzymes and proteins such as creatine kinase (CK) (Peake et al., 2017). This myofibrillar disruption leads to functional loss of muscle strength and power and increase in delayed-onset muscle soreness (DOMS). The EIMD seems to be related to the intensity of exercise and muscle contractions at 80 and 100% of maximal voluntary contraction (MVC) as well as exercise with pronounced eccentric muscle actions produce large muscle damage than exercise protocols using lighter loads, such as 40 and 60% of MVC (Chen et al., 2007).

Resistance exercise (RE) combined with blood flow restriction (BFR) is a training method that consists in applying pressure cuffs placed in the proximal region of the limbs which are inflated to a set pressure throughout exercise (Loenneke et al., 2013). BFR training has been studied as an exercise strategy for patients who are contra-indicated from performing traditional heavy-load resistance exercise. BFR training can induce skeletal muscle strength and hypertrophy even in the presence of lower muscle damage (Franz et al., 2017, 2018).

Few studies, however, have analyzed changes in the acute leukocyte profile associated with muscle damage in resistance exercise with BFR. Thus, the aim of this study was to analyze the acute effects of high intensity resistance exercise and low intensity resistance exercise combined with BFR on leukocyte profile and its association with EIMD. Our study hypothesis is that RE+BFR would induce less leukocytosis due to the lower EIMD compared to the RE group alone.

## Materials and Methods

### Experimental Approach to the Problem

The participants visited the laboratory six times for data collection. At the first visit, the participants were familiarized with 1RM testing procedure, BFR and the vertical jump performance test. At the second visit (after 48 h) anthropometric measurements and one-repetition maximum test were obtained. In the third visit (after 48 h), it was determined the blood flow restriction pressure and retest the maximum repetition. The fourth visit was 7 days after the third one. In the fourth visit the participants performed the resistance exercise protocol. The fifth and sixth (each one after 24 h) visit consisted of performing only blood collections and measures of muscle damage. The present study used a randomized design to perform the exercise protocol.

### Participants

Twenty healthy men volunteers (Table 1), with aged from 18 to 36 years, who had been involved in regular RE for at least 1 year and at least 3 days per week, were enrolled in this study. Sample size was determined using GPower 3.1 software with a statistical power of 80% and medium effect size of 0.15. The following exclusion criteria were adopted: (i) use of drugs that could affect cardiorespiratory responses; (ii) bone-, joint- or muscle-diagnosed problems that could limit the execution of elbow flexor; (iii) systemic hypertension (≥140/90 mmHg or use of antihypertensive medication); (iv) metabolic disease; and (v) use of exogenous anabolic–androgenic steroids, toxic drugs or medication with potential effects on physical performance.

**TABLE 1:** Subjects’ main characteristics at baseline.

Groups	N	Age (years)	Height (cm)	BM (kg)	BMI (kg/m^2^)
RE80	10	23.9 ± 5.2	167 ± 10	66.5 ± 11.5	23.6 ± 1.9
RE40 + BFR	10	26.0 ± 6.8	168 ± 8	69.8 ± 12.4	24.5 ± 3.2

All procedures and risks were explained to participants before they provided written consent to participate. This study was approved by the Ethics Committee Federal University of Espirito Santo. Participants were instructed to refrain from strenuous activities at least 72 h before the RE sessions and to avoid the use of any pain-relieving and anti-inflammatory drugs and to maintain their normal food intake and lifestyle habits throughout the study.

### Exercise Protocols

The volunteers arrived at the lab between 8 and 8:30 am. Participants performed one of two different interventions on bilateral leg press exercise equated by total work (sets × repetitions × load): high intensity RE (RE80, *n* = 10): three sets of with 80% of 1RM until concentric muscle failure; and low intensity with blood flow restriction RE (RE40+BFR, *n* = 10): three sets of 25 repetitions with 40% of one repetition maximum (1RM) combined with 80% BFR. The number of repetitions in the RE40+BFR was calculated based on the total work achieved by the RE80 group (Table 2). Each group performed the leg press exercise with cadence fixed at 2 s at each concentric/eccentric muscle actions with 1 min of rest between sets.

**TABLE 2:** Blood flow restriction and total work.

Groups	Blood flow restriction pressure (%)	Total blood flow restriction pressure (mmHg)	Total work (kg)
RE80	–	–	10,284 ± 3,695
RE40 +BFR	80	220 ± 39	11,576 ± 2,908

### Determination of the Blood Flow Restriction Pressure

Subjects were asked to lie on a supine position while resting comfortably. A vascular Doppler probe (DV-600, Martec, Ribeirão Preto, SP, Brazil) was placed over the tibial artery to determine the BFR pressure (mmHg). A standard blood pressure cuff (width 18 cm; length 35 cm) attached to the proximal portion of thigh was inflated up to the point in which the auscultatory pulse of the tibial artery was interrupted. The BFR pressure was maintained constant throughout the exercise session. The cuff pressure used during the training protocol was determined as 80% of the necessary pressure for complete blood flow occlusion in a resting condition (Laurentino et al., 2012).

### One-Repetition Maximum Test

The procedures adopted for 1RM test for the bilateral leg press exercise (Sickert, Brazil) were followed the recommendations described by Brown (Brown and Weir, 2001). In the first set, participants performed eight repetitions with a load correspondent to 50% of their estimated 1RM obtained during the familiarization session. In the second set, they performed three repetitions with 70% of their estimated 1RM. A 2-min interval was allowed between warm-up sets. After the completion of second set, participants rested for 3 min and then had up to five attempts to achieve their 1RM with 3-min interval enforced between attempts. The 1RM strength on the leg press exercise was recorded and reproduced throughout the study. Tests were conducted by an experienced researcher, and strong verbal encouragement was provided during the attempts.

### Blood Lactate Concentration

After local cleansing of middle finger, participant’s finger was lanced, and the capillary blood sample was collected using heparinized capillary tubes. The blood lactate concentration was determined with an electrochemical device (YSI 1500 Select; Yellow Springs, OH, United States).

### Rating of Perceived Exertion and Pain

Immediately after each set, subjects were asked to report their rating of perceived exertion (RPE) and pain (RPP) using Borg’s 6–20 scale (Vieira et al., 2015; Neto et al., 2016).

### Blood Collection

Approximately 5 mL of blood samples were collected from the antecubital vein in vacutainer tubes containing EDTA in the moments before, immediately, 24 and 48 h after each exercise bout. Blood samples were centrifuged at 1,500 *g* for 10 min at 4°C and the serum was stored at −80°C for subsequent analysis for leukocytes, neutrophils, lymphocytes, monocytes, using a blood analyzer (Beckman Coulter T660; Beckman Coulter, Inc., Miami, Florida).

### Indirect Markers of Muscle Damage

#### CK Levels Assay

The CK levels were carried in an automated biochemical analyzer Bioclin2200 using commercially available kits (Bioclin, Belo Horizonte, Brazil) following the manufacturer’s specifications.

#### cfDNA Concentration Assay

A standard curve with seven concentrations was generated by serial dilution of commercial salmon sperm DNA (Sigma-Aldrich). The curve was evaluated in triplicate resulting a standard curve used to calculate the DNA concentrations. cfDNA concentrations were directly analyzed with a fluorescent nuclear stain (SYBR Gold) in serum samples. Briefly, SYBR Gold (1: 10,000 dilutions in PBS) was added to serum in 96-well black plates and fluorescence was recorded using a fluorometer Varioskan Flash (Thermo Fisher Scientific, Inc., Rockford, IL, United States) with an excitation wavelength of 485 nm, and emission wavelength of 535 nm (Goldshtein et al., 2009).

#### Vertical Jump Performance Test

Squat jump (SJ) and countermovement jump (CMJ) tests. For each protocol three jumps were performed with 30 s of interval between jumps. The SJ was carried out from a squatting position, with an approximately 90° of knee flexion and the hands fixed on the hip. This position was maintained for 3 s and then, upon verbal command, they jumped vertically to maximum height. No countermovement was allowed. In the CMJ the participants started from an erect standing position with knees fully extended (knee = 180°). Upon the verbal command, made a downward countermovement approximately to the same starting position as the SJ (knee = 90°) and then jumped vertically for maximum height in one continuous movement. A contact mat was used to perform (Jump Test-Hidrofit, Brazil). All the subjects had been previously familiarized with SJ and CMJ tests.

### Statistical Analysis

Values were expressed as the mean ± standard deviation (SD) for all variables. Data were analyzed for normality (Gaussian distribution) using the Shapiro-Wilk test. As data were normally distributed, parametric analyzes were used. Statistical analyses were performed by two-way ANOVA to evaluate differences between trials and time-points. When the ANOVA showed a significant interaction effect, a Tukey’s *post hoc* test was used to locate differences between variables. In addition, unpaired *t*-tests were used to analyze the differences between groups on total work. The statistical analyses were performed using Prism software (Prism 6, GraphPad Software, Inc., San Diego, CA, United States). A value of *p* < 0.05 was regarded as statistically significant.

In addition, the effect size (ES) and confidence interval 95% (CI) was used to verify the magnitudes of changes between assessments of the protocols as trivial (0–0.19), small (0.20–0.49), medium (0.50–0.79), large (0.80–1.29) and very large (1.30 or greater) (Rosenthal, 1996).

## Results

**FIGURE 1:**
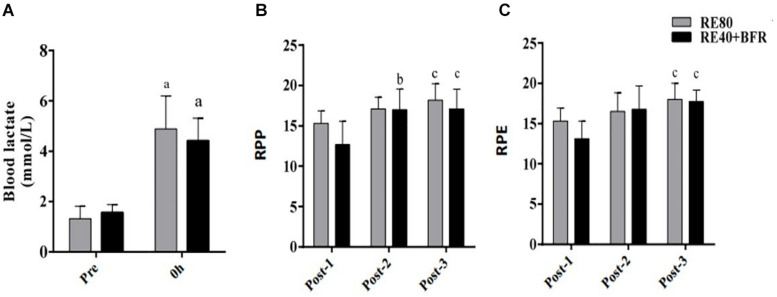
Blood lactate concentration **(A)**, rating of perception pain **(B)**, and rating of perception exertion **(C)**. Data are shown by mean ± SD. *a* = *p* < 0.05 to both RE80 and RE4O+BFR vs. Pre, *b* = *p* < 0.05 Post-2 vs. Post-1 and *c* = *p* < 0.05 Post-3 vs. Post-1.

The Figure 1A shows blood lactate concentration after RE40+BFR and RE80. Blood lactate increased ∼3-fold compared to resting values in both groups. In spite of the statistically significant increase in blood lactate concentration after exercise (*p* < 0.0001) no differences were observed between the groups (RE40+BFR, 4.4 ± 1.0 vs. RE80, 4.8 ± 1.3, mmol/L) groups.

For RPP the two-way ANOVA indicated significant interaction [*F*(2,36) 5.894; *p* = 0.0061] and main factor for time [*F*(2,36) 56.07; *p* < 0.0001] without significant main effect for exercise condition [*F*(1,18) 2.011; *p* = 0.1733]. RPP increased in set 2 and 3 in the RE40+BFR group, while in RE80 only in set 3 (Figure 1B). For RPE the two-way ANOVA indicated no significant interaction [*F*(2,36) 3.145; *p* = 0.0551] or main factor for exercise condition [*F*(1,18) 0.9464; *p* = 0.3435]. There was, however, a significant main effect for time [*F*(2,36) 25.94; *p* < 0.0001]. RPE increased only at set 3 in both groups compared to set 1 (Figure 1C).

**FIGURE 2:**
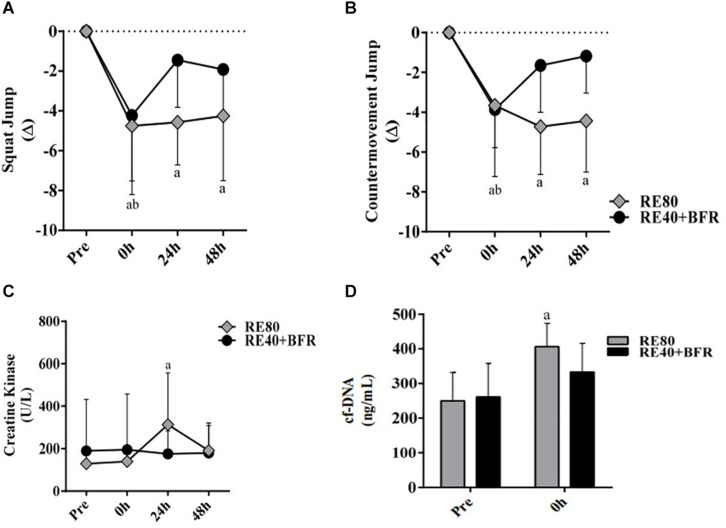
EIMD markers. Squat jump performance **(A)**, countermovement jump performance **(B)**, Creative kinase **(C)** and Cell-free DNA **(D)**. The data are expressed as the mean ± SD. *a* = *p* < 0.05 RE80 compared with pre, and *b* = *p* < 0.05 to RE4O+BFR vs. Pre.

For SJ the two-way ANOVA indicated significant interaction [*F*(3,54) 2.874; *p* = 0.0445] and main factor for both time [*F*(3,54) 18.85; *p* < 0.0001] without significant main effect for exercise condition [*F*(1,18) 0.1301; *p* = 0.7226]. SJ performance (Figure 2A) decreased significant immediately after exercise in both groups compared to pre-exercise values. However, only in RE80 the performance reduction persisted until 48 h after exercise.

For CMJ the two-way ANOVA indicated significant interaction [*F*(3,54) 5,571; *p* = 0.0021] and main factor for time [*F*(3,54) 17.44; *p* < 0.0001] without significant main effect for exercise condition [*F*(1,18) 0.02198; *p* = 0.8838]. The CMJ performance (Figure 2B) had the same response of SJ, with a reduction in jump height immediately after exercise in both groups but still impaired after 48 h only in RE80.

For CK the two-way ANOVA indicated significant interaction [*F*(3,54) 3.469; *p* = 0.0227] and main factor for time [*F*(3,54) 7.119; *p* < 0.0001] without significant main effect for exercise condition [*F*(1,18) 1.518; *p* = 0.2347]. Serum CK activity was statistically different only in RE80 group post-24 h compared to pre-values, without changes in the RE40+BFR group (Figure 2C). cfDNA (Figure 2D) increased significantly only in RE80 group immediately after exercise (406.3 ± 67.2, ES 0.80, CI −0.14–1.68) compared with pre (249.6 ± 82.2, ES 2.08, CI 0.92–3.07), without meaningful changes in the RE40+BFR group (*p* = 0.11).

**FIGURE 3:**
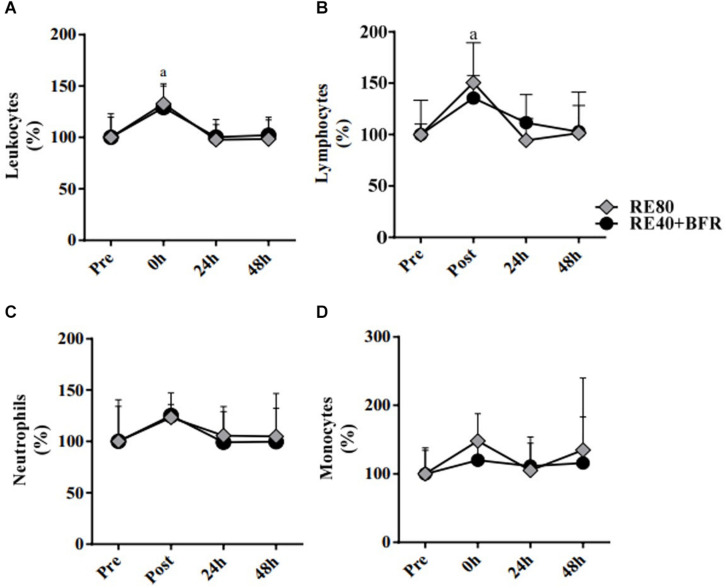
White blood cells. Leukocytes **(A)**, Lymphocytes **(B)**, Neutrophils **(C)**, and Monocytes **(D)**. The data are expressed as the mean ± SD. *a* = *p* < 0.05 both RE4O+BER and RE80 compared with pre.

The Figure 3 shows the leukocytes profile response induced by the exercise protocols. For leukocytes the two-way ANOVA indicated no significant interaction [*F*(3,54) 1.317; *p* = 0.2785] or main factor for exercise condition [*F*(1,18) 3,906; *p* = 0.0637]. Only main effect for time [*F*(3,54) 26.78; *p* < 0.0001] was observed. It can be observed that the total leukocytes increased immediately after exercise (10.07 ± 2.67 × 10^3^/mm^3^) compared with pre-exercise (7.59 ± 1.48, ES 1.12, CI 0.14–2.01) in RE80. The same was observed in the group RE40+BFR: immediately after exercise (8.25 ± 0.96) vs. pre-exercise (6.57 ± 1.50, ES 1.33, CI 0.31–2.24) (Figure 3A). Regarding lymphocytes, the same pattern was observed (Figure 3B). The two-way ANOVA indicated no significant interaction [*F*(3,54) 1.79; *p* = 0.1601] or main factor for exercise condition [*F*(1,18) 0.6513; *p* = 0.4302]. Only main effect for time [*F*(3,54) 14.92; *p* < 0.0001] was observed. RE80 immediately after exercise (3.65 ± 1.31) vs. RE80 pre-exercise (2.48 ± 0.83, ES 1.11, CI 0.13–2.00) and RE40+BFR immediately after exercise (3.03 ± 0.65) vs. RE40+BFR pre-exercise (2.22 ± 0.23, ES 1.76, CI 0.67–2.71). However, no changes in neutrophils (Figure 3C): RE80 pre vs. immediately after exercise (4.30 ± 1.47 vs. 5.34 ± 2.07, ES 0.56, CI –0.36–1.42) and RE40+BFR pre vs. immediately after exercise (3.72 ± 1.51 vs. 4.43 ± 1.23, ES 0.51, CI −0.40–1.38), and monocytes (Figure 3D): RE80 pre vs. immediately after exercise (0.48 ± 0.18 vs. 0.71 ± 0.30, ES 0.93, CI −0.03–1.81) and RE40+BFR pre vs. immediately after exercise (0.37 ± 0.13 vs. 0.44 ± 0.15, ES 0.50, CI −0.41–1.37) were observed. There were no differences in the leukocyte profile in the post-exercise times 24 and 48 h in relation to the pre-exercise, as well as in the profiles of lymphocytes, neutrophils, and monocytes.

## Discussion

The aim of this study was to analyze the acute effects of high intensity resistance exercise and low intensity resistance exercise combined with BFR on leukocyte profile associated with EIMD. The main findings of our study were as follows: (1) there were no differences in the blood leukocyte profile of the RE40 + BFR group compared to the RE group, despite the lower EIMD in the RE40 + BFR, (2) both groups showed leukocytosis and lymphocytosis immediately after exercise, (3) leukocytosis and lymphocytosis was greater in the RE40 + BFR group which may help to explain the fast recovery after EIMD.

Historically, inflammation was recognized as a phenomenon that compromised post-exercise recovery. It is now accepted that the inflammatory responses occurs related to muscle damage and plays an important role in its regeneration and recovery (Peake et al., 2005). Mechanical and metabolic stress associated with EIMD, activates several types of cells in order to recover and remodel the injured muscle (Peake et al., 2017). Among these various cell types are the inflammatory cells such as lymphocytes, neutrophils and monocytes (Hyldahl and Hubal, 2014). Interestingly and unexpectedly, this smaller increase was accompanied by a larger effect size on lymphocytosis and leukocytosis, however, with no difference in effect size in relation to neutrophils.

The accumulation of leukocytes in the inflamed muscle is a gradual process that depends on the extent of the damage (Hyldahl et al., 2014). Studies report the presence of leukocytes in the muscle in response to moderate to severe muscle damage, usually induced by maximum eccentric exercise (Peake et al., 2017). There are several mechanisms by which these cells participate in the repair and regeneration of the damaged muscle. Neutrophils and macrophages act in the removal of cellular debris through phagocytosis and production of reactive species (Nguyen and Tidball, 2003; Arnold et al., 2007). Understanding the phenotypic transfer of pro-inflammatory macrophages (M1) to anti-inflammatory macrophages (M2) is essential, since M1 macrophages interact with the proliferation of satellite cells, while M2 macrophages participate in the differentiation of these satellite cells in addition to the synthesis of connective tissue (Tidball and Villalta, 2010; Schlagheck et al., 2020).

Although research has focused more on neutrophils and macrophages, recent studies have demonstrated a predominant role of lymphocytes in the regeneration process, cells that until then had been linked only to pathological muscle processes. Lymphocytes participate in the muscle repair process in two basic ways: regulate myogenic cell activity and regulate muscle immune cell infiltrate. CD8+ T-cells facilitate the expression of C-C motif chemokine ligand 2 (CCL2) by macrophages residing in the muscle, essential for the recruitment of pro-inflammatory monocytes in the injured muscle (Zhang et al., 2014). Regulatory T cells (Treg) support muscle regeneration through the expression of amphiregulin growth factor (AREG) (Burzyn et al., 2013). Burzyn et al. (2013) demonstrated that treatment with AREG normalizes the evolution of the muscle transcriptome throughout the muscle repair process and promotes myogenic differentiation *in vitro* (Burzyn et al., 2013). The absence or deficiency of Tregs in the muscles after injuries is related to decreased fiber growth and failure in phenotypic change from M1 to M2 and, therefore, exaggeration in the inflammatory process (Burzyn et al., 2013; Kuswanto et al., 2016). The main subpopulation responsible for the largest effect size also present in leukocytosis in the RE40 + BFR group was lymphocytes. therefore, we believe that lymphocytes are related to faster muscle recovery in the RE40 + BFR group compared to the RE80 group.

There are two mechanisms related to acute post-exercise Leukocytosis: (1) increased cardiac output and, consequently, blood flow in the pulmonary, hepatic and splenic vascular bed, which induces, through shear stress, leukocyte demargination; and (2) increase in the expression of β-2 adrenergic and glucocorticoids receptors in leukocytes, thus increasing their activation in response to adrenaline and glucocorticoids during exercise [13]. The greater activation of the sympathetic-adrenal-medullary axis accompanied by greater release of catecholamines that occurs in exercises with blood flow restriction (Spranger et al., 2015), may be the mechanism that explains the greater size of lymphocytosis effect observed in the RE40 + BFR group, facilitating thus the sequestration of these cells by the injured muscle.

Muscle function and performance are markers of EIMD (Peake et al., 2017). Although CK was different between conditions, both groups showed reduction in the vertical jump performance immediately after RE. Thus, we believe that the mechanism of reduced performance is different. Classically, RE cause EIMD by mechanical stress which is thought to represent the primary factor in muscle adaptive response (Tidball and Villalta, 2010) and explain the CK increase after 24 h. However, studies have shown that the reduction in exercise performed after BFR has been associated with decreased oxygen supply and increased metabolic stress (Downs et al., 2014). Since our results showed an increase in blood lactate immediately after exercise in the RE+BFR group we believe that the metabolic stress could explain the reduction in performance immediately after exercise.

Another interesting data observed in our study was the response of cfDNA, we and other have already demonstrated that it may be new marker of EIMD or a predictor of exercise performance 24 h after the exercise session (Atamaniuk et al., 2010; Andreatta et al., 2018). The second mechanism is reinforced by our data since cfDNA did not increased significantly immediately after exercise in the RE40+BFR group while exercise performance was already recovered 24 h after exercise. In addition, Tug et al. (2015) showed in 2015 that the majority of cfDNA released during aerobic exercise was derived from the hematopoietic system. One possible hypothesis could be the release of cfDNA from neutrophil, in a mechanism called neutrophil extracellular traps (NETs) (Brinkmann, 2004). Immediately after one aerobic exercise session shows increased NET-like structures in the blood (Beiter et al., 2014). However, both raised points require further investigation in future studies to deeply elucidate the contribution of different cell type in release cfDNA during both aerobic as well as strength exercise.

A limitation of this study is the fact that the leukocyte profile was analyzed only immediately after exercise and at times 24 and 48 h after. However, for a deeper understanding of the leukocyte profile dynamics it would be interestingly to evaluate every 2–4 h. Also, it should be emphasized that only indirect markers of muscle EIMD were used in this study.

## Conclusion

In summary, RE associated with BFR was accompanied by a greater leukocytosis and lymphocytosis immediately after exercise, with no difference in neutrophils. This leukocyte blood profile may be related to less muscle damage, as well as faster muscle recovery 24 and 48 h post-exercise. The results of the present study may have useful practical application, both in sports and clinical settings. The use of RE + BFR can be a valid alternative to promote gains in muscle mass and strength, imposing less overload on the joints and promoting faster recovery in muscle function between training sessions. It can, therefore, be an interesting strategy in the clinical environment for patients with functional limitations, and even for athletes during periods of high training volume, promoting improved recovery between training sessions and competitions.

## Data Availability Statement

All datasets presented in this study are included in the article/supplementary material.

## Ethics Statement

This study was approved by the Ethics Committee Federal University of Espírito Santo. The patients/participants provided their written informed consent to participate in this study.

## Author Contributions

LS: discussion and manuscript writing. MA, VC, WM, and LF: protocols and data collection. VB: research orientation, discussion, and manuscript writing. All authors contributed to the article and approved the submitted version.

## Conflict of Interest

The authors declare that the research was conducted in the absence of any commercial or financial relationships that could be construed as a potential conflict of interest.
